# Long-Term Survival Effect of the Interval between Postoperative Chemotherapy and Radiotherapy in Patients with Completely Resected Pathological N2 Non-Small-Cell Lung Cancer

**DOI:** 10.3390/cancers13102494

**Published:** 2021-05-20

**Authors:** Shih-Min Lin, Hsiu-Ying Ku, Che-Yu Hsu, Chih-Liang Wang, Gee-Chen Chang, Cheng-Shyong Chang, Tsang-Wu Liu

**Affiliations:** 1 Department of Radiation Oncology, Chang Gung Memorial Hospital at Linkou, Taoyuan 333, Taiwan; 8905024@cgmh.org.tw; 2 National Institute of Cancer Research, National Health Research Institute, Miaoli 350, Taiwan; shiuo@nhri.org.tw; 3 Department of Healthcare Administration, Asia University, Taichung 413, Taiwan; 4 Division of Radiation Oncology, Department of Oncology, National Taiwan University Hospital, Taipei 100, Taiwan; 101495@nuth.gov.tw; 5 National Taiwan University Cancer Center, National Taiwan University and Academia Sinica, Taipei 100, Taiwan; 6 Graduate Program of Data Science, National Taiwan University and Academia Sinica, Taipei 100, Taiwan; 7 Department of Thoracic Medicine, Chang Gung Memorial Hospital, Taoyuan 333, Taiwan; wang@adm.cgmh.org.tw; 8 College of Medicine, Chang Gung University, Taoyuan 333, Taiwan; 9 School of Medicine and Institute of Medicine, Chung Shan Medical University, Taichung 402, Taiwan; cshy1888@csh.org.tw; 10 Division of Pulmonary Medicine, Department of Internal Medicine, Chung Shan Medical University Hospital, Taichung 402, Taiwan; 11 Division of Hematology and Oncology, Chang Bing Show Chwan Memorial Hospital, Changhua 505, Taiwan; cs4816@gmail.com

**Keywords:** postoperative radiotherapy, postoperative chemotherapy, NSCLC, pN2, IMRT

## Abstract

**Simple Summary:**

In patients with completely resected stage III pN2 non-small cell lung cancer (NSCLC), adjuvant chemotherapy of 4–6 cycles was recommended prior to post-operative radiotherapy (PORT). However, some were given concurrently or early-sequentially with PORT. The objectives of this study were to verify the benefit of adjuvant sequential chemotherapy and radiotherapy (SCRT) relative to that of concurrent chemoradiotherapy (CCRT) in an Asian population and to identify the optimal timing of initiation of PORT as part of adjuvant SCRT. A longer interval (>104 days and <180 days) between the initiation of adjuvant chemotherapy and PORT was associated with improved OS compared with CCRT. No locoregional recurrence-free survival (LRFS) difference related to the interval between the initiation of adjuvant chemotherapy and PORT was observed. In older patients (aged >60 years), the benefit of delayed PORT initiation was more significant. We suggest that PORT should be postponed in the completed-resected pN2 elderly patients.

**Abstract:**

(1) Purpose: To investigate the effects of the time interval between initiation of adjuvant chemotherapy and radiotherapy on survival outcomes in patients with completely resected stage IIIA pN2 non-small-cell lung cancer (NSCLC); (2) Methods: Data on 2515 patients with completely resected stage IIIA pN2 NSCLC in 2007–2017 were extracted from the Taiwan Cancer Registry Database. The survival outcomes in patients who underwent concurrent chemoradiotherapy (CCRT) and sequential chemotherapy and radiotherapy (SCRT) with either a short (SCRT1) or long (SCRT2) interval between treatments were estimated using Kaplan–Meier, Cox regression, and propensity score matching (PSM); (3) Results: Multivariate analyses of OS showed that SCRT2 (hazard ratio [HR] 0.64, *p* = 0.017) was associated with improved overall survival (OS). After PSM, the median OS periods were 64 and 75 months in the SCRT1 and SCRT2 groups, respectively, which differed significantly from that of 58 months in the CCRT group (*p* = 0.003). In elderly patients, SCRT2 significantly improved survival relative to CCRT before PSM (*p* = 0.024) and after PSM (*p* = 0.002); (4) Conclusions: A longer interval between initiation of adjuvant chemotherapy and postoperative radiotherapy (PORT; SCRT2) improved OS relative to CCRT; the benefits were greater in elderly patients (age >60 years).

## 1. Introduction

Non-small-cell lung cancer (NSCLC) is the leading cause of cancer-related mortality worldwide [1]. For stage IIIA-pN2 NSCLC, surgical resection followed by adjuvant chemotherapy is the mainstay of treatment [2,3]. However, the role of postoperative radiotherapy (PORT) as part of multimodal therapy for completely resected IIIA pN2 NSCLC remains controversial. Its benefit has been a subject of debate since a meta-analysis of data from 2128 patients enrolled in nine randomized trials addressed its adverse effects in early-stage pN1 NSCLC [4]. Several subsequent studies were conducted to evaluate the effect of PORT in terms of improvement of locoregional control and overall survival (OS) [5,6,7,8,9,10,11]. Due to lack of strong evidence supporting the use of PORT for completely resected pN2 NSCLC, its use declined from 65% in 1992 to 37% in 2002 [5].

Ideal timing of PORT initiation also remains controversial. Adjuvant chemotherapy of 4–6 cycles was recommended prior to PORT, however, some were given concurrently or early-sequentially with PORT [12,13]. Two retrospective studies conducted in Asia demonstrated the effectiveness of early PORT, which benefited the OS in patients with stage IIIA pN2 NSCLC when followed by or administered concurrently with postoperative chemotherapy (POCT) [12,13]. However, the sequential chemotherapy and radiotherapy (SCRT) was associated with improved OS compared with adjuvant concurrent chemoradiotherapy (CCRT) by previous Adjuvant Navelbine International Trialist Association (ANITA) subgroup analyses, which demonstrated the benefit of PORT following adjuvant chemotherapy in patients with pN2 disease [14]. Most recently, the LungART trial, which focused on completely-resected pN2 disease, released its preliminary report of reduced evidence on the efficacy of PORT [15]. Adjuvant radiotherapy commenced within 4–8 weeks of surgery and SCRT were both included in the LungART trial [15]. Decisions regarding the optimal timing of PORT initiation must be made with balanced consideration of need for disease control by adequate adjuvant chemotherapy and possible reduction of the locoregional benefit of PORT. In the setting of SCRT for patients with completely resected IIIA pN2 NSCLC, this timing remains a subject of debate.

The genetic makeup of tumors differs between Caucasian and Asian patients. For example, sensitizing epidermal growth factor receptor (*EGFR*) mutations are found in approximately 10% of Caucasian patients compared with up to 50% of Asian patients with NSCLC [16]. The effects of PORT administered as parts of adjuvant CCRT and SCRT need to be examined in large-scale studies conducted in Asian populations.

The objectives of this study were to verify the benefit of adjuvant SCRT relative to that of CCRT in an Asian population and to identify the optimal timing of initiation of PORT as part of adjuvant SCRT in patients with completely resected stage III pN2 NSCLC. To our knowledge, this nationwide population-based study involves the largest cohort where the majority underwent intensity-modulated radiotherapy (IMRT) to evaluate the effect of interval between postoperative chemotherapy and radiotherapy.

## 2. Materials and Methods

### 2.1. Data Source and Study Population

Data on patients with NSCLC that was newly diagnosed between 1 January 2007 and 31 December 2017 were extracted from the Taiwan Cancer Registry Database (TCRD), a nationwide database of oncology outcomes that captures the data from 97% of all newly diagnosed cancer cases in Taiwan [17]. The TCRD dataset includes clinical information and contains detail radiotherapy information not available in other Taiwan National Health Insurance Research Dataset (NHIRD). The follow-up period was extended from the index date, defined as the date of NSCLC diagnosis, to 31 December 2018. Survival during this period was examined via linkage to death certificates registered in the National Death Database. Our institute’s review board approved the study protocol (EC1070305-E). The information on informed patient consent waived due to the retrospective nature of this study. From this dataset, data on patients with non-metastatic pN2 NSCLC who underwent microscopically negative-margin (R0) resection and at least lobectomy, adjuvant chemotherapy, and PORT were included. To minimize treatment variability, we excluded data of patients who received PORT doses <45 Gy and those who started adjuvant chemotherapy >90 days after surgery.

To evaluate the impact of PORT timing on OS, the patient cohort was divided into the CCRT (first cycle of chemotherapy administered within 14 days of PORT initiation), SCRT1 (first cycle of chemotherapy administered 15–103 days before PORT), and SCRT2 (first cycle of chemotherapy administered 104–180 days before PORT) groups. The median interval between the first chemotherapy cycle and PORT in the SCRT1 and SCRT2 groups was 103 days. The maximum interval of 180 days accommodated PORT initiation up to 8 weeks after six cycles of chemotherapy, allowing some delay between chemotherapy cycles. Patients who initiated chemotherapy 14 days after PORT initiation were excluded from the study. In addition, we excluded those who were lost to follow-up or died within 3 months of diagnosis. Patients with no disease recurrence who were followed for <3 months after PORT were excluded from the CCRT group to avoid immortal time bias.

Data on the following patient characteristics were collected: age, sex, year of diagnosis, treatment facility type, surgery type, Eastern Cooperative Oncology Group (ECOG) performance status (PS), smoking habit, tumor grade, histology, tumor size, tumor location, pathological T stage, pathological N stage, surgical margin status, radiation treatment time, status of target therapy usage, and total radiation dose. *EGFR* mutation information was not available in the TCRD until 2011. Information on the primary endpoint of OS, defined as the period from the index time of diagnosis to the date of death, was obtained from the TCRD and the Ministry of the Interior database.

### 2.2. Statistical Analysis

Analysis of variance and chi-square (*X*^2^) test were used to evaluate inter-group differences in continuous and categorical variables, respectively. Univariate and multivariate Cox proportional-hazard modeling with hazard ratio (HR) calculation was used to identify factors associated with locoregional recurrence-free survival (LRFS), distant metastasis-free survival (DMFS), and OS. Such models were also employed to examine associations beween groups and the survival outcome while controlling for clinical (e.g., smoking, tumor size, and histology) and demographic (e.g., ECOG PS) variables. These variables represented significant predictors of survival in univariate and multivariate analyses. OS, LRFS, and DMFS were estimated using Kaplan–Meier analysis, and differences therein were assessed using the log-rank test. All tests were two tailed, and *p* < 0.05 was considered to represent statistical significance. Propensity score matching (PSM) was used to account for differences in baseline patient characteristics among treatment groups. Matching was performed based on patient characteristics and disease factors, including age, sex, tumor size, surgery type, treatment facility type, tumor site, and treatment time, using the method described by Rosenbaum and Rubin [18]. All calculations were performed using SAS version 9.3 (SAS Institute Inc., Cary, NC, USA) and SPSS version 22.0 (SPSS Inc., Chicago, IL, USA) software.

## 3. Results

### 3.1. Patient Selection and Characteristics

**Figure 1:**
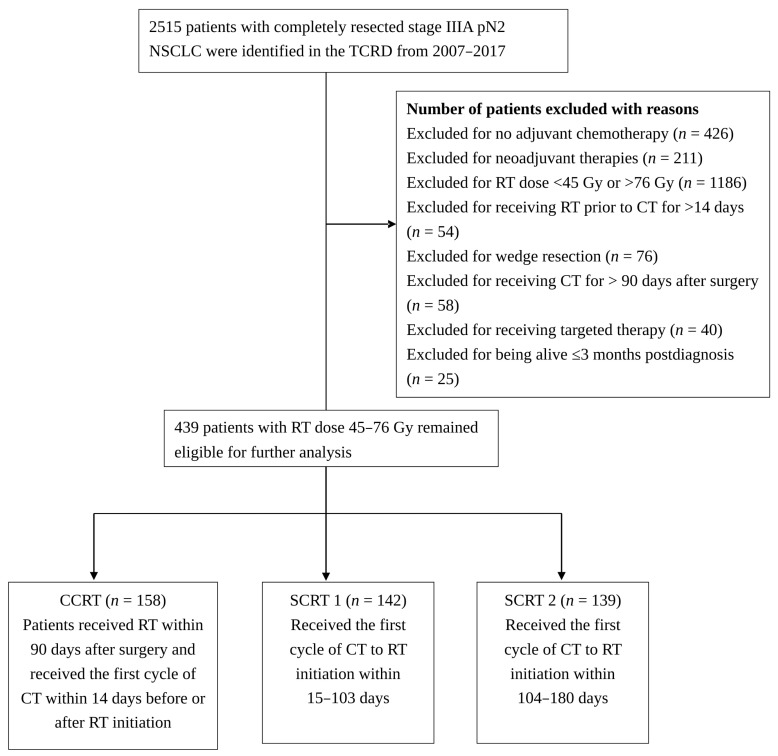
Flow chart representing the selection of completely resected pN2 NSCLC patients over the course of the study. Abbreviations: CCRT: concurrent chemoradiation; CT: chemotherapy; NSCLC: non-small-cell lung cancer; RT: radiotherapy; SCRT1: sequential chemoradiation group 1; SCRT2: sequential chemoradiation group 2; TCRD: Taiwan Cancer Registry Database.

In total, 2515 patients with completely resected stage IIIA pN2 NSCLC were identified in the TCRD. Patients who underwent neoadjuvant chemotherapy, or chemoradiation, or other pre-operative therapy were excluded from our study. After exclusion of those not given adjuvant CCRT or SCRT, 439 patients remained eligible for further analysis (Figure 1). The cohort was divided into CCRT, SCRT1, and SCRT2 groups; demographic characteristics are summarized by group in Table 1. Sixty-four percent of patients with completely resected stage IIIA pN2 disease received SCRT after PORT, of whom 142 and 139 patients were assigned to the SCRT1 and SCRT2 groups, respectively. The most common histological diagnosis was adenocarcinoma (*n* = 344, 78%), and most patients were treated after 2010 and received PORT at a dosage of 45–55 Gy, delivered as intensity-modulated radiation therapy (IMRT). No significant difference was observed among the three groups in the distribution of histology types (*p* = 0.633), year of diagnosis (*p* = 0.816), ECOG PS (*p* = 0.567), sex (*p* = 0.882), smoking habit (*p* = 0.168), tumor site (*p* = 0.325), tumor size (*p* 0.595), *EGFR* mutation status (*p* = 0.297), or PORT dose (*p* = 0.415). More patients in the CCRT group had well- to moderately differentiated tumors (*p* < 0.001) and received IMRT (*p* = 0.025). Medical centers adopted SCRT more frequently than did regional hospitals (*p* = 0.033).

### 3.2. Factors Associated with Patient Survival

**Figure 2:**
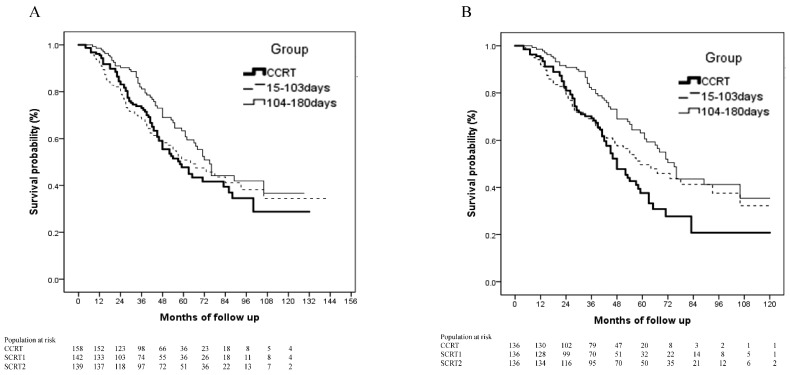
Kaplan–Meier survival curves of OS in patients treated according to three PORT schedules (CCRT, SCRT1 [15–103 days], SCRT2 [104–180 days]). (**A**) Before PSM, log-rank test, *p* = 0.095; CCRT vs. SCRT1, *p* = 0.94; CCRT vs. SCRT2, *p* = 0.037; and (**B**) After PSM, log-rank test, *p* = 0.003; CCRT vs. SCRT1, *p* = 0.188; CCRT vs. SCRT2, *p* < 0.01). Abbreviations: CCRT: concurrent chemoradiation; OS: overall survival; PORT: postoperative radiotherapy; PSM: propensity score matching; SCRT1: sequential chemoradiation group 1; SCRT2: sequential chemoradiation group 2.

**Figure 3:**
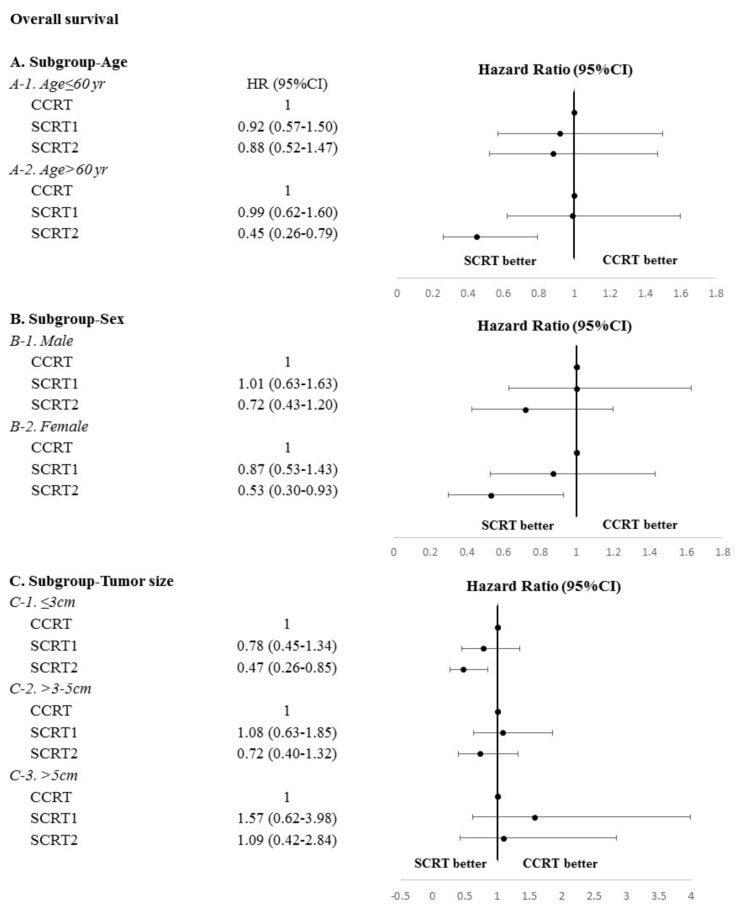
Forest plots of aHRs showing the effect of PORT timing on OS. The 95% CIs are also shown. Abbreviations: aHR: adjusted hazard ratio; CCRT: concurrent chemoradiation; OS: overall survival; PORT: postoperative radiotherapy; SCRT1: sequential chemoradiation group 1; SCRT2: sequential chemoradiation group 2; CIs: confidence intervals.

The median OS duration for the entire cohort was 67 (interquartile range, 58.4–75.6) months. In univariate analyses, age <60 years, female sex, small tumors, treatment at medical centers, and SCRT2 were significantly associated with improved OS (Table 2). Multivariate analyses adjusted for covariates showed that (HR 1.02, *p* = 0.016), treatment at a small facility (HR 1.41, *p* = 0.019), and tumor size >5 cm (HR 2.5, *p* < 0.001) were significantly associated with increased risk of mortality, whereas SCRT2 (HR 0.64, *p* = 0.017) and tumor location in the lower lung lobe (HR 0.71, *p* = 0.037) were associated with improved OS (Table 2). The median OS durations in the CCRT, SCRT1, and SCRT2 groups were 58 (95% confidence interval [CI], 45.2–70.8), 64 (95% CI, 44.5–83.5), and 75 (95% CI, 67.4–82.5) months, respectively (*p* = 0.095; Figure 2A). Forest plots showed that age >60 years (adjusted hazard ratio [aHR] 0.45, *p* = 0.005), female sex (aHR 0.53, *p* = 0.028), and tumor size <3 cm (aHR 0.47, *p* = 0.013) were associated with decreased mortality in the SCRT2 group (Figure 3). After adjustment for confounders, the effect of SCRT2 on survival lost statistical significance in patients aged <60 years (aHR 0.88, *p* = 0.615) and in male patients (aHR 0.72, *p* = 0.203; Figure 3). Multivariate Cox regression analysis revealed no significant difference in LRFS between the SCRT1 and SCRT2 groups and the CCRT group (HR 0.83, *p* = 0.0646 and HR 0.96, *p* = 0.924, respectively) or DMFS (HR 0.88, *p* = 0.563 and HR 0.84, *p* = 0.443, respectively; Supplemental Figure S1A,B). Notably, the SCRT2 group showed a significant DMFS benefit relative to the CCRT group (aHR 0.46, *p* < 0.01; Supplemental Figure S1B).

### 3.3. Impact of Interval between Post-Operative Chemotherapy and Radiotherapy on Survival

**Figure 4:**
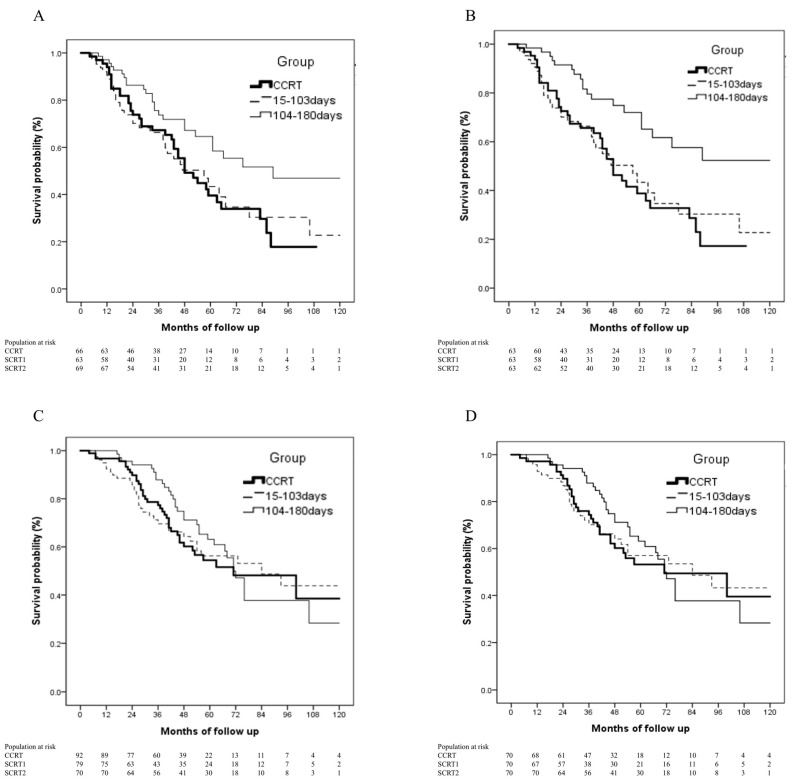
Kaplan–Meier OS curves in patients treated according to the three PORT schedules (CCRT, SCRT1 [15–103 days], SCRT2 [104–180 days]) in subgroups stratified by age. (**A**) Age >60 years, before PSM, log-rank test, *p* = 0.024; (**B**) Age >60 years, after PSM, log-rank test, *p* = 0.002; (**C**) Age ≤60 years, before PSM, log-rank test, *p* = 0.856; (**D**) Age ≤60 years, after PSM, log-rank test, *p* = 0.871. Abbreviations: CCRT: concurrent chemoradiation; OS: overall survival; PORT: postoperative radiotherapy; PSM: propensity score matching; SCRT1: sequential chemoradiation group 1; SCRT2: sequential chemoradiation group 2.

After PSM, data from 408 patients were available for analysis (Figure 2B). Demographic and cancer characteristics were well balanced among the three groups. The median OS durations in the SCRT1 and SCRT2 groups were 64 (95% CI, 43.1–84.9) and 75 (95% CI, 67.4–82.5) months, respectively, which differed significantly from the median OS duration of 58 (95% CI, 44.6–71.4) months in the CCRT group (log-rank test, *p* = 0.003; Figure 2B). Elderly patients in the SCRT2 group had significantly better survival than did those in the CCRT group before PSM (log-rank test, *p* = 0.024; Figure 4A), and this survival advantage remained significant after PSM (log-rank test, *p* = 0.002; Figure 4B). No such survival benefit was observed in younger patients before (log-rank test, *p* = 0.856; Figure 4C) or after (log-rank test, *p* = 0.871; Figure 4D) PSM.

## 4. Discussion

This study investigated the impact of a longer interval between adjuvant chemotherapy and PORT on the prognosis in patients with completely resected stage IIIA pN2 NSCLC. Crude 5-year OS proportions in the CCRT, SCRT1, and SCRT2 groups were 42%, 48%, and 62%, respectively, and were comparable to OS values obtained in retrospective studies on PORT and POCT administration in patients with stage IIIA N2 disease [12,13,19,20].

Among the patients with completely resected IIIA pN2 NSCLC, most recurrent tumors were located outside of the surgical area and accounted for most mortalities. Several randomized controlled trials have shown that adjuvant chemotherapy plays a key role in prolonging disease-free survival and OS [3,21,22]. However, high locoregional recurrence rates of 20–40% have been reported, even after adjuvant chemotherapy for completely resected IIIA pN2 NSCLC [14,21,23]. Consistent with the hypothesis that PORT improves locoregional control, which would translate to an OS benefit, retrospective studies of NCDB data have demonstrated that modern PORT at adequate dosages was associated with better OS in patients with completely resected IIIA pN2 NSCLC (5-year OS, 27.8% vs. 34.1%; *p* < 0.001) [8,9]. Furthermore, studies based on NCDB data have found that the survival outcome was associated with the timing of PORT, with better 5-year OS observed in patients treated with SCRT than in those treated with adjuvant CCRT for completely resected stage IIIA pN2 disease [19,20]. The work by Francis et al. based on NCDB data supports the detrimental effect of adjuvant CCRT relative to SCRT for completely resected IIIA pN2 NSCLC (median OS duration, 32.5 vs. 58.8 months; *p* < 0.001) [20]. In another NCDB data analysis, Moreno et al. found that the median OS duration was significantly improved in patients undergoing SCRT compared with those undergoing CCRT (53 vs. 37 months, *p* < 0.001) [19]. Although the influence of the sequencing of adjuvant chemotherapy and RT in patients with completely resected NSCLC has been investigated, [12,13,19,20] the optimal sequencing schedule, and especially the timing of PORT as part of SCRT, remains a subject of debate.

Among trials conducted to evaluate the efficacy of adjuvant chemotherapy in patients with stage III N2 NSCLC, the International Adjuvant Lung Trial, in which three or four cycles of cisplatin-based adjuvant chemotherapy were administered, demonstrated a 5-year survival benefit of 4.1% (HR 0.86; 95% CI, 0.76–0.98; *p* = 0.03) [3]; the ANITA trial, in which four cycles of adjuvant cisplatin were administered in combination with vinorelbine, demonstrated an absolute 5-year survival benefit of 8.6% [21]; and Ou et al. [22] administered four cycles of vinorelbine/carboplatin or paclitaxel/carboplatin doublet adjuvant chemotherapy and demonstrated an absolute survival advantage of 12.0% at 5 years. The common duration of the four cycles of adjuvant chemotherapy was 12 weeks, and the timing of PORT initiation was 2–3 weeks after the completion of chemotherapy. The cut-off point for SCRT2 in our study accommodated the completion of the four cycles of adjuvant chemotherapy and subsequent PORT (84 days + 20 days). The DMFS benefit observed in the SCRT2 group may reflect the greater probability of completing a course of adjuvant chemotherapy, which translates to improve OS with no detrimental effect on LRFS, which can occur with delayed PORT initiation.

Subgroup analyses in the present study showed that elderly patients who received SCRT2 benefited the most and had significantly improved survival compared with those who received CCRT, before and after PSM. Generally, younger patients have a greater capacity to tolerate surgery, subsequent chemotherapy, and PORT. In previous studies conducted in Asian populations, early PORT (concurrent with or followed by chemotherapy) had an OS benefit in patients with stage IIIA pN2 NSCLC [12,13], and younger age (mean <60 years) might help to maintain the locoregional OS benefit. In contrast, several recent studies conducted with NCDB data, most of which examined cohorts with mean ages >60 years, yielded results demonstrating the importance of postponing PORT until after chemotherapy completion [19,20]. Our study produced similar results, showing that older patients benefited from a longer interval between the initiation of adjuvant chemotherapy and PORT. Compared with the most recent results of LungART, our entire cohort has a favorable 3-year OS, 75% (our PORT cohort) versus 66.5% (PORT arm of LungART), and 68.5% (no PORT arm of LungART) [15]. First, the LungART trial allowed adjuvant radiotherapy began within surgery 4-8 week, which means allowed early PORT [15]. Second, the mean age of LungART was 61 years old [15]. According to our finding, the elderly would not get survival advantage if they took early PORT. In the LungART trial, the cardiopulmonary toxicity was supposed to overwhelm the benefits of mediastinal relapse-free survival [15]. There was 3D conformal radiotherapy technique adoption in LungART [15], however, there were only 17% of patients who underwent 3D-conformal radiotherapy in our study. The majority of patients in our cohort underwent IMRT, and modern technique would be necessary to lower surrounding normal organs toxicity [9,24]. In previous NCDB analysis by Corso et al., there were only 17% who used IMRT, whereas others used 3D-conformal radiotherapy [8]. The 5-year OS was 34.1% in NCDB and 53% in our study (TCRD) [8]. It is necessary to deliver adjuvant radiotherapy safer, instead of suspending usage. Our study showed the long-term survival effects of different intervals between adjuvant chemotherapy and radiotherapy on the basis of routine modern PORT adoption.

This study has several limitations. Data of chemotherapy regimens and number of cycles were not recorded in the TCRD. However, the practice patterns of chemotherapy were examined in recent years by other Taiwan National Health Insurance Research Dataset (NHIRD) [25,26]. According to the study of Liang et al., platinum-based doublet chemotherapy was provided to the majority of the patients (66.9%), and it was combined with gemcitabine (33.8%) [26]. The second and third most common regimens were vinorelbine alone (13.0%) and platinum with docetaxel (11.6%) [26]. Our study period was conducted from 2007 to 2017, and the frequency of using platinum with pemetrexed was supposed to be high in patients with adenocarcinoma, owing to a longer OS than that in patients who received other platinum-based regimens [26,27,28,29]. Targeted therapies are providing survival benefits to *EGFR* mutant NSCLC disease as shown in much recent evidence that is emerging [30,31]. However, we excluded patients with targeted therapy from our study. Additionally, this study performed a retrospective analysis of non-randomized data without reporting the patients’ safety data, and although we used PSM to account for confounders among the covariates examined, confounding by unmeasured covariates may have persisted. For example, some patients in the CCRT and SCRT1 groups may have received suboptimal chemotherapy.

Despite these limitations, our study has several strengths. Our cohort used IMRT in the majority, compared to a previous NCDB study that used IMRT in only 17% [8]. Such modern radiotherapy factors would lower the treatment-related mortality associated with PORT [7,9,24,32,33]. IMRT is beneficial in node-positive disease compared with 3D-CRT [24]. To our knowledge, this study is the largest cohort study using IMRT modern techniques to N2 patients. The TCRD is a population-based database, and our results can be generalized to other cohorts. In addition, locoregional and distant recurrence events are registered in the TCRD, enabling more detailed analysis. To our knowledge, this study is the first to demonstrate the OS benefit of delayed PORT initiation after the administration of adjuvant chemotherapy for completely resected IIIA pN2 NSCLC, especially among older patients, in an Asian population. In addition, the availability of information on recurrence events across subgroups in the population helped us determine whether delayed PORT initiation after adjuvant chemotherapy had a negative impact on LRFS, and to identify SCRT2 subgroups with better DMFS (Supplemental Figure S1B).

## 5. Conclusions

In the context of postoperative treatment for completely resected stage IIIA pN2 NSCLC, a longer interval (>104 days and <180 days) between the initiation of adjuvant chemotherapy and PORT was associated with improved OS compared with CCRT. No LRFS difference related to the interval between the initiation of adjuvant chemotherapy and PORT was observed. In older patients (aged >60 years), the benefit of delayed PORT initiation was more significant. We suggest that PORT should be postponed in the completely-resected pN2 elderly patients.

## Figures and Tables

**Table 1:** Clinical and demographic characteristics in patients stratified by PORT schedule (CCRT, SCRT1, and SCRT2).

Variables	CCRT		SCRT1		SCRT2		*p* Value
	*n*	%	*n*	%	*n*	%	
Sex							0.882
Male	77	48.7	72	50.7	71	51.1	
Female	81	51.3	70	49.3	68	48.9	
Age at diagnosis, years							
Mean ± SD	57.42 ± 10.75		58.54 ± 10.13		60.55 ± 9.37		0.029
Year of diagnosis							0.816
2007–2010	35	22.2	33	23.2	28	20.1	
2011–2017	123	77.8	109	76.8	111	79.9	
Facility type							0.033
Regional hospital	71	44.9	69	48.6	47	33.8	
Medical center	87	55.1	73	51.4	92	66.2	
Surgery							0.718
Lobectomy	152	96.2	137	96.5	133	95.7	
Pneumonectomy	2	1.3	0	0.0	1	0.7	
Segmental resection	4	2.5	5	3.5	5.	3.6	
Histology							0.633
Adenocarcinoma	120	75.9	117	82.4	107	77.0	
SqCC	19	12.0	12	8.5	16	11.5	
ASC	4	2.5	4	2.8	7	5.0	
Others	15	9.5	9	6.3	9	6.5	
Grade (differentiation)							<0.001
Well, moderately	87	55.1	72	50.7	70	50.4	
Poorly	49	31.0	65	45.8	68	48.9	
Undifferentiated and unknown	22	13.9	5	3.5	1	0.7	
Tumor size (cm)							0.595
≤3	69	43.7	62	44.0	62	44.6	
>3–5	70	44.3	56	39.7	52	37.4	
>5	19	12.0	23	16.3	25	18.0	
Pathologic T stage							0.072
I	35	22.2	47	33.1	29	20.9	
II	101	63.9	84	59.2	91	65.5	
III	22	13.9	11	7.7	19	13.7	
Tumor site							0.325
Upper lobe	73	46.2	78	54.9	80	57.6	
Middle lobe	17	10.8	15	10.6	8	5.8	
Lower lobe	65	41.1	48	33.8	50	36.0	
Central region	3	1.9	1	0.7	1	0.7	
RT technique							0.025
2D and 3D	18	11.4	33	23.2	24	17.3	
IMRT	140	88.6	109	76.8	115	82.7	
Radiation dose (cGy)							
4500–5500	115	72.8	99	69.7	107	77.0	0.415
5501–6000	28	17.7	27	19.0	25	18.0	
>6000	15	9.5	16	11.3	7	5.0	
RT treatment time							
Mean ± SD	41.1 ± 6.4		39.8 ± 6.6		38.0 ± 4.5		<0.001
*EGFR* mutation status							0.297
Wild type	38	24.1	35	24.7	24	17.3	
Mutation	31	19.6	34	23.9	34	24.5	
Unknown	89	56.3	73	51.4	81	58.2	
ECOG scale of performance status							0.567
0–1	112	70.9	93	65.5	100	72.0	
≥2	3	1.9	3	2.1	1	0.7	
Unknown	43	27.2	46	32.4	38	27.3	
Smoking habit							0.168
Non-smoker	79	50.0	68	47.9	61	43.9	
Smoker	27	17.1	16	11.3	27	19.4	
Quit smoking	17	10.8	25	17.6	23	16.6	
Unknown	35	22.1	33	23.2	28	20.1	
Median follow time (months)							
Median [IQR]	42.0 (25.5–58)		38.0 (20–60)		48.0 (33–72)		0.029

Abbreviations: ASC: adenosquamous cell carcinoma; CCRT: concurrent chemoradiation; ECOG: The Eastern Cooperative Oncology Group; *EGFR*: epidermal growth factor receptor; IMRT: intensity-modulated radiation therapy; IQR: interquartile range; RT: radiotherapy; SD: standard deviation; SCRT1: sequential chemoradiation group 1; SCRT2: sequential chemoradiation group 2; SqCC: squamous cell carcinoma.

**Table 2:** Univariate and multivariate analyses of overall survival using Cox proportional hazards modeling.

Variables	Univariate		Multivariate	
	HR (95% CI)	*p* Value	HR (95% CI)	*p* Value
Group				
CCRT	1		1	
SCRT1	0.98 (0.70–1.37)	0.906	0.97 (0.69–1.36)	0.850
SCRT2	0.71 (0.50–1.00)	0.050	0.64 (0.44–0.92)	0.017
Sex				
Male	1		1	
Female	0.75 (0.57–0.99)	0.046	0.91 (0.68–1.22)	0.520
Age at diagnosis (year)				
≤60	1		1	
>60	1.37 (1.04–1.81)	0.027	1.02 (1.00–1.03)	0.016
Year of diagnosis				
2007–2010	1			
2011–2017	0.79 (0.58–1.07)	0.129		
Facility Type				
Medical center	1		1	
Regional hospital	1.31 (0.99–1.73)	0.059	1.41 (1.06–1.89)	0.019
Surgery				
Segmental resection	1		1	
Lobectomy	1.71 (0.55–5.34)	0.359	1.53 (0.48–4.92)	0.473
Pneumonectomy	4.81 (0.80–28.84)	0.086	3.05 (0.47–19.85)	0.244
Histology				
Adenocarcinoma	1			
SqCC	1.06 (0.67–1.70)	0.795		
ASC	0.93 (0.41–2.10)	0.856		
Others	0.63 (0.33–1.19)	0.152		
Grade (differentiation)				
Well and moderately	1			
Poorly	0.84 (0.62–1.12)	0.233		
Undifferentiated and unknown	0.71 (0.97–1.35)	0.297		
Tumor size (cm)				
≤3	1		1	
>3–5	1.14 (0.83–1.55)	0.423	1.18 (0.86–1.63)	0.308
>5	2.26 (1.55–3.29)	<0.001	2.50 (1.68–3.73)	<0.001
Pathologic T stage				
I	1			
II	1.23 (0.88–1.72)	0.226		
III	1.44 (0.87–2.38)	0.153		
Tumor site				
Upper lobe	1		1	
Middle lobe	1.22 (0.77–1.95)	0.399	1.36 (0.85–2.19)	0.202
Lower lobe	0.75 (0.55–1.02)	0.063	0.71 (0.51–0.98)	0.037
Central region	1.87 (0.59–5.90)	0.286	1.61(0.47–5.48)	0.447
Radiotherapy technique				
2D+3D	1			
IMRT	1.06 (0.75–1.50)	0.735		
Radiation dose (cGy)				
4500–5500	1			
5501–6000	0.78 (0.53–1.14)	0.200		
>6000	0.95 (0.57–1.59)	0.842		
RT treatment time	1.01(0.98–1.03)	0.631	0.99 (0.969–1.018)	0.573
*EGFR* mutation status				
Wild type	1			
Mutation	0.76 (0.48–1.21)	0.245		
ECOG scale of performance status				
0–1	1			
≥2	4.89 (1.98–12.10)	0.001		
Smoking habit				
Non-smoker	1			
Smoker	0.87 (0.55–1.38)	0.550		
Quit smoking	1.43 (0.93–2.19)	0.100		

Abbreviations: ASC: adenosquamous cell carcinoma; CCRT: concurrent chemoradiation; *EGFR*: epidermal growth factor receptor; HR: hazard ratio; IMRT: intensity-modulated radiation therapy; RT: radiotherapy; SCRT1: sequential chemoradiation group 1; SCRT2: sequential chemoradiation group 2; SqCC: squamous cell carcinoma; CI: confidence interval.

## Data Availability

The data presented in this study are available on request from the corresponding author. The data are not publicly available due to restriction of privacy and ethical policy.

## Supplementary Materials

The following are available online at https://www.mdpi.com/article/10.3390/cancers13102494/s1, Figure S1: Forest plots of aHRs showing the effect of PORT timing on (A) local regional recurrence-free survival, and (B) distant metastasis-free survival.

## Abbreviations

CI	confidence interval
CIs	confidence intervals
aHR	adjusted hazard ratio
HR	hazard ratio
ASC	adenosquamous cell carcinoma
CCRT	concurrent chemoradiation
ECOG	The Eastern Cooperative Oncology Group
*EGFR*	Epidermal growth factor receptor
IMRT	intensity-modulated radiation therapy
IQR	interquartile range
RT	radiotherapy
SD	standard deviation
SCRT1	sequential chemoradiation group 1
SCRT2	sequential chemoradiation group 2
SqCC	Squamous cell carcinoma
TCRD	Taiwan Cancer Registry Database
NSCLC	non-small-cell lung cancer
OS	overall survival
PORT	postoperative radiotherapy
PSM	propensity score matching
