# Supplementary material for: Long-Term Survival Effect of the Interval between Postoperative Chemotherapy and Radiotherapy in Patients with Completely Resected Pathological N2 Non-Small-Cell Lung Cancer
Source: Cancers (Basel). 2021 May 20;13(10):2494. doi: 10.3390/cancers13102494 (PMC8160867; doi:10.3390/cancers13102494)
Supplement: Supplementary file 1 [file cancers-13-02494-s001.zip › cancers-1169839-supplementary.pdf]

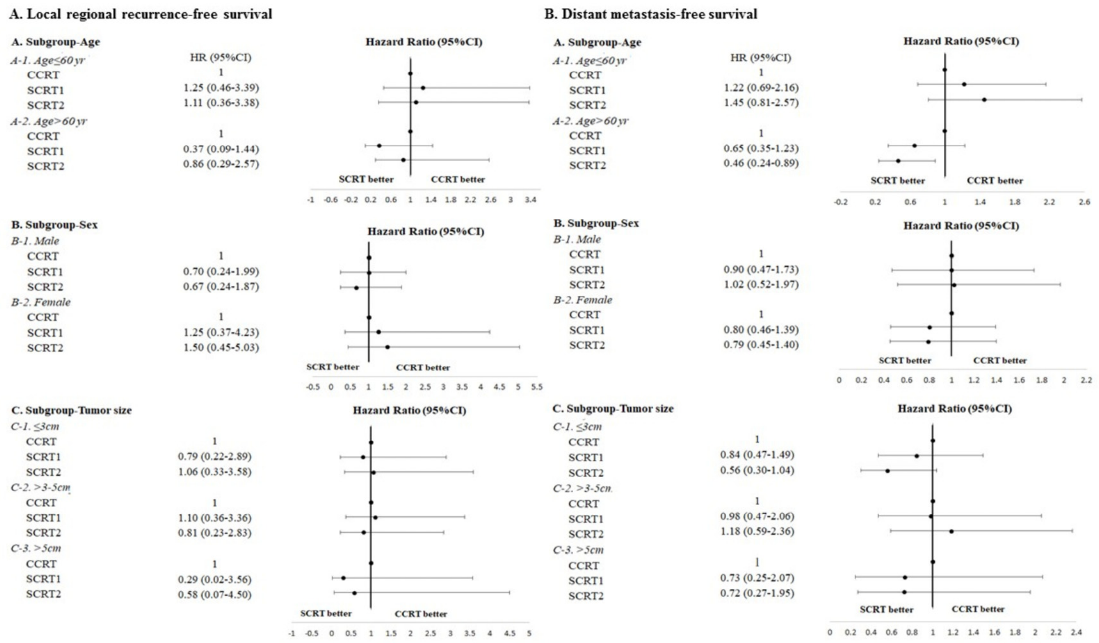

Supplementary Figure S1: Forest plots of aHRs showing the effect of PORT timing on (**A**) local regional recurrence-free survival, and (**B**) distant metastasis-free survival. The 95% CIs are also shown. *Abbreviations*: aHR: adjusted hazard ratio; CCRT: concurrent chemoradiation; PORT: postoperative radiotherapy; SCRT: sequential chemoradiation; CIs: confidence intervals.
